# A rare case of CD20^−^ primary cutaneous diffuse large B-cell lymphoma, leg type

**DOI:** 10.1016/j.jdcr.2024.08.036

**Published:** 2024-09-28

**Authors:** Juliette M. Kersten, Anouschka N. Lenderink, Koen D. Quint, Rosanne Ottevanger

**Affiliations:** Department of Dermatology, Leiden University Medical Centre, Leiden, The Netherlands

**Keywords:** B-cell lymphoma, CD20, CD20^**−**^, diffuse large B-cell lymphoma, leg type, primary cutaneous B-cell lymphoma, primary cutaneous diffuse large B-cell lymphoma

## Introduction

Primary cutaneous diffuse large B-cell lymphoma, leg type (PCDLBCL, LT) is rare disease with an unfavorable prognosis and is the most aggressive type of cutaneous B-cell lymphoma.^1^, ^2^, ^3^ The diagnosis is characterized by erythematous tumors frequently presenting on the lower portion of the leg(s) and the absence of extracutaneous manifestations upon initial diagnosis, distinguishing it from the comparatively more prevalent systemic variant known as diffuse large B-cell lymphoma (DLBCL). Histologically both PCDLBCL, LT and DLBCL are characterized by a monomorphous proliferation of centroblasts and/or immunoblasts. In contrast, immunohistochemical profiling for DLBCL can lack expression of the B-cell lineage marker CD20, this has not been described in current literature for PCDLBCL, LT.^4^ In patients with PCDLBCL, LT, dissemination to extracutaneous sites occurs frequently, often proposing a challenge for accurate diagnosis.^5^ Opportunely, the introduction of the anti-CD20 monoclonal antibody, rituximab, has drastically improved the prognosis for patients with CD20^+^ cutaneous B-cell lymphoma.^6^ This report presents a remarkable case of recurrent, CD20^**−**^ PCDLBCL, LT in a male patient, whose treatment proved to be challenging as, because of the lack of CD20^+^ cells, conventional treatment including rituximab could not be prescribed.

## Case report

**Fig 1:**
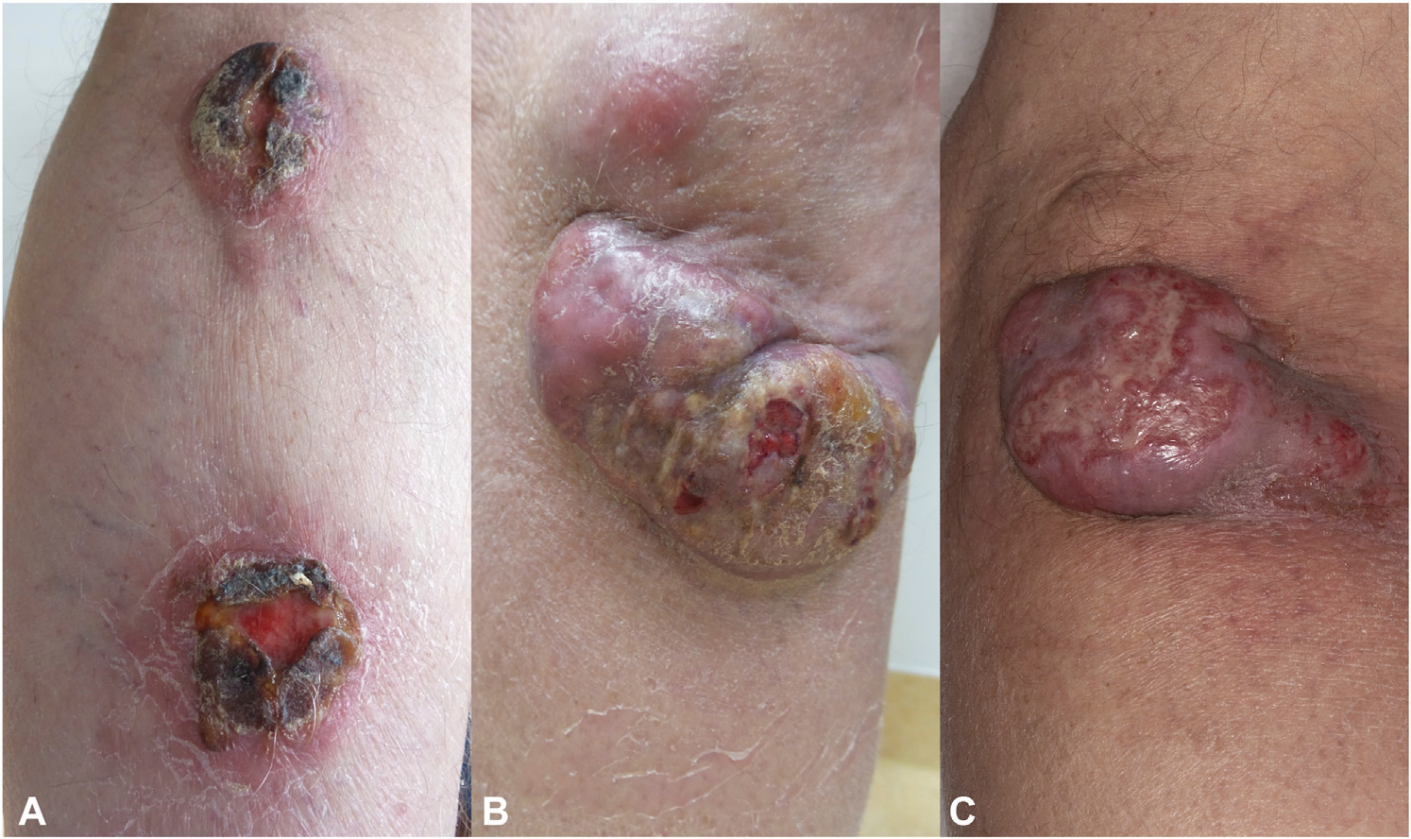
Clinical presentation at different time points. **A,** September 2021: first presentation at Dermatology Department. Multiple tumors on the medial side the lower portion of the left leg. **B,** May 2022: new localizations of tumors on the lateral side of the left knee. **C,** June 2022: subtle reduction in tumor size after treatment with bendamustine and polatuzumab vedotin.

**Fig 2:**
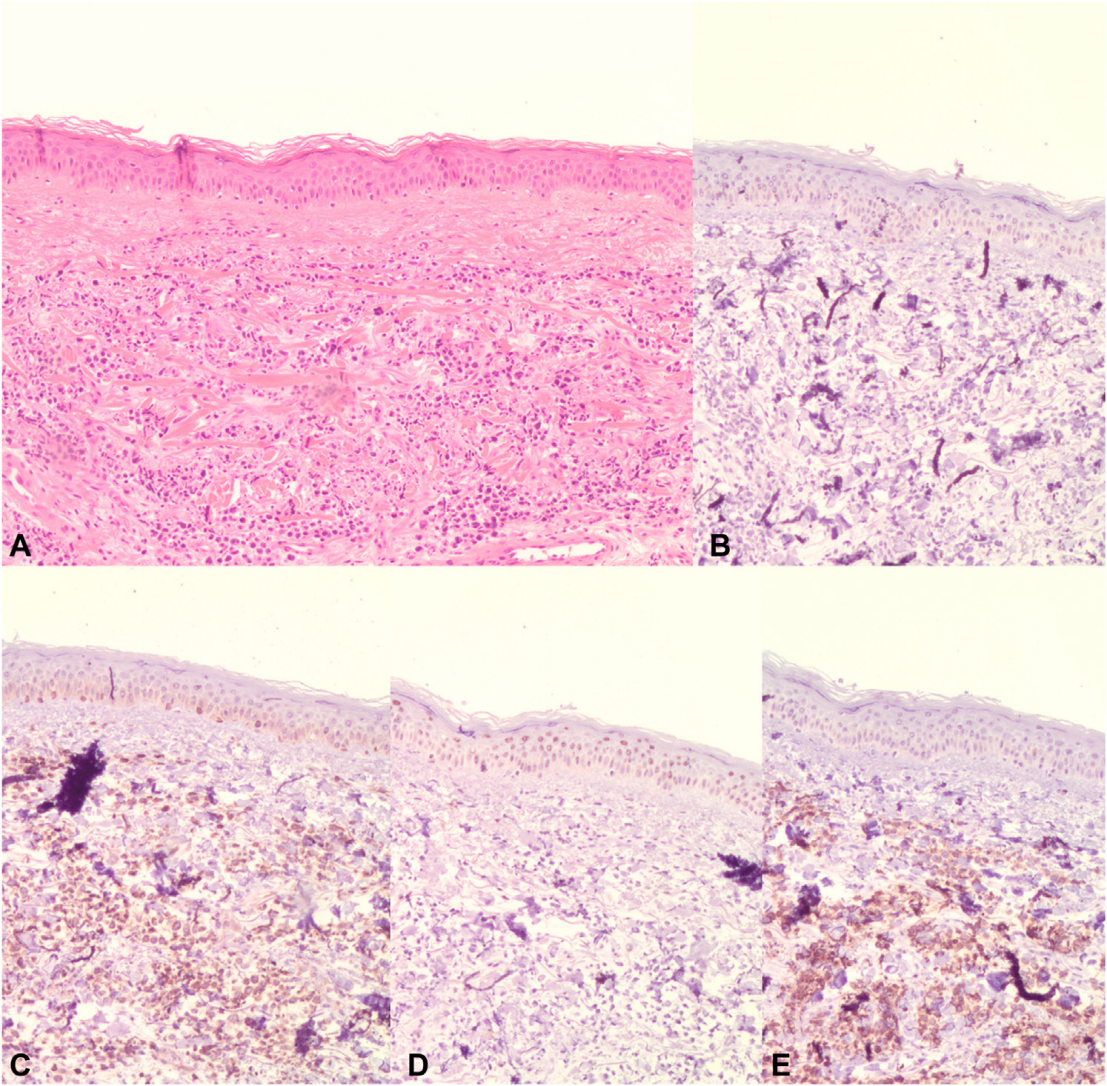
**A,** Biopsy detail showing a dense dermal infiltrate of atypical lymphocytes. **B,** Details of CD20^**−**^ of abnormal lymphoid cells in the dermis. **C,** Details of strong diffuse BCL2 expression of atypic lymphocytes in the dermis. **D,** Details of negativity of abnormal lymphoid cells in the dermis. **E,** Details of strong diffuse CD79 expression of atypic lymphocytes in the dermis. (**A,** Hematoxylin-eosin stain; original magnifications: **A-E,** ×100.)

A 79-year old man, without a relevant medical history, presented with 2 tumors on the leg at the outpatient clinic of the Leiden University Medical Center. The lesions on the left leg had developed in the last 3 months and were asymptomatic (Fig 1). No enlarged lymph nodes were discovered during physical examination. Furthermore, skin biopsies, blood examination, and a positron emission tomography-computed tomography (PET-CT) were performed. The immunohistochemical analysis revealed positivity for CD79a and MUM-1, with heterogeneous expression of MYC. The markers CD20, PAX5, immunoglobulin M, BCL6, and CD138 were negative. Histologically, the biopsy specimen showed a kappa-monotypic blastoid proliferation with a very high Ki-67 proliferation index (>90%). No MYC, BCL2, or BCL6 rearrangements were detected by fluorescence in situ hybridization. Next-generation sequencing identified a class 5 pathogenic variant in TP53 (exon 5). Differential diagnosis initially considered a blastic plasmacytoid dendritic cell neoplasm because of the weakly positive CD123. However, the absence of kappa restriction, along with negative staining for CD56, CD4, and CD68, ruled out blastic plasmacytoid dendritic cell neoplasm. Therefore, the favored diagnosis is large B-cell lymphoma with immunoblastic features. Clinicopathologic correlation combined with no blood involvement and no internal abnormalities on the PET-CT resulted in the diagnosis of PCDLBCL, LT. Remarkably, immunohistochemical staining was negative for CD20 (Fig 2).

Because of the absence of CD20^+^ B-cell markers in the tumor, the patient was not eligible for the recommended treatment regimen for PCDLBCL, LT consisting of rituximab combined with cyclophosphamide, hydroxydaunorubicin, vincristine (Oncovin) and prednisone according to the World Health Organization-European Organization for Research and Treatment of Cancer, as rituximab by nature is an anti-CD20 monoclonal antibody and therefore not able to target CD20^**−**^ tumor cells.^7^^,^^8^ Accordingly, a CHOP regimen was administered, excluding rituximab, for 6 cycles. Treatment evaluation by PET-CT showed complete metabolic remission of the cutaneous tumors. Unfortunately, within 1 month after the last dose of systemic treatment, new lesions developed on the patient’s lower portion of the left leg. A subsequently performed skin biopsy showed an immunohistochemical profile identical to the one obtained <6 months ago. This confirmed the recurrence of the CD20^**−**^ PCDLBCL, LT.

**Fig 3:**
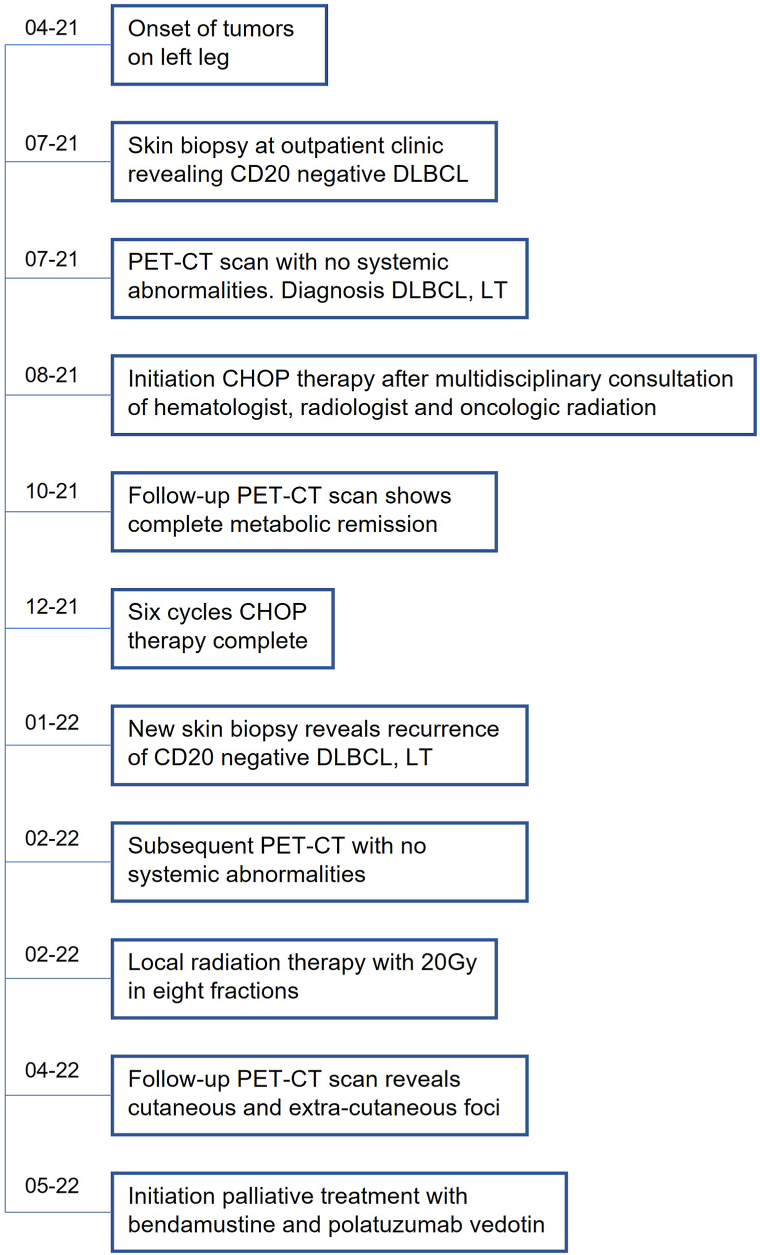
Timeline. *CHOP*, Cyclophosphamide, hydroxydaunorubicin, vincristine (Oncovin), and prednisone; *DLBCL*, diffuse large B-cell lymphoma; *DLBCL, LT*, diffuse large B-cell lymphoma, leg type; *Gy*, gray; *PET-CT*, positron emission tomography-computed tomography; *RT*, radiation therapy.

A follow-up PET-CT scan was performed, which showed no extracutaneous disease (Fig 3). Because of the rapid recurrence of the cutaneous disease, the patient was treated with 8 photon-radiotherapy sessions of 2.5 Gy, a cumulative dose of 20 Gy. Complete clinical remission of the tumors was achieved. Within 2 weeks after the final irradiation, the disease progressed and new lesions developed on his leg just at the border of the radiation field, clinically suspect for a second recurrence of the PCDLBCL, LT. A PET-CT revealed recurrence of the lymphoma with new, hypermetabolic cutaneous foci located near the knees, as well as extracutaneous foci located in multiple lymph nodes.

Because of the extent of the disease and its aggressive course, after multidisciplinary consultation and shared decision making, palliative radiotherapy was initiated, followed by 2 cycles of bendamustine and polatuzumab vedotin. After the second course of treatment, therapy was discontinued because of cutaneous tumor progression, and the patient passed away within 2 months.

## Discussion

According to the World Health Organization-European Organization for Research and Treatment of Cancer, PCDLBCL, LT is a rare subtype of cutaneous B-cell lymphoma and is associated with a poor prognosis.^1^^,^^2^ Systemic DLBCL is considerably more prevalent than PCDLBCL, LT and has different clinical characteristics, in contrast to similar histopathologic findings. Notably, varieties of CD20^**−**^ DLBCL, including plasmablastic lymphoma, primary effusion lymphoma, anaplastic lymphoma, kinase-positive DLBCL, and large B-cell lymphoma, have been described.^4^ These subtypes often exhibit a higher frequency of the nongerminal center B-cell phenotype, which is associated with poorer prognosis and treatment response.^9^ Alternative regimens to CHOP, such as cyclophosphamide, vincristine, doxorubicin, methotrexate alternating with ifosfamide, etoposide, and cytarabine (CODOX-M/IVAC) and infusional etoposide, vincristine, doxorubicin, cyclophosphamide and prednisone (EPOCH/CHOEP) have been proposed as potential treatment options, but there is still no established golden standard of care for (systemic) CD20^**−**^ B-cell lymphomas. To our knowledge, this is the first case describing the therapeutical challenge of a patient with CD20^**−**^ PCDLBCL, LT. This remarkable case presentation proposes a challenge regarding the disease management, because of the inability to treat the patient with the standard care treatment including the potent monoclonal antibody rituximab. Genomic analysis in previous literature has revealed that up to 40% of DLBCL, LT cases have recurrent genetic alterations in PD-L1/PD-L2, presenting a hopeful future possibility for PD-1 checkpoint inhibitors in this rare population.^10^

A timely and accurate diagnosis is essential to provide the patient with prompt and aggressive treatment. Clinicians should be aware of the possibility of CD20^**−**^ in PCDLBCL, LT. Furthermore, clinicians must be attentive to the follow-up, and be alert that frequent PET-CT monitoring should be initiated, even after initial successful aggressive multichemotherapy.

Future research should focus on understanding the significant factors driving these types of aggressive tumors to rapid recurrence and dissemination, as a means of better targeting the cells driving this process.

## Conflicts of interest

None disclosed.
